# Natural flavonoids in peptic ulcer disease: preclinical evidence for gastroprotection versus healing of established ulcers

**DOI:** 10.3389/fphar.2026.1950919

**Published:** 2026-10-28

**Authors:** Ibtihal Segmani, Fatima Mourabiti, Houda Filali, Imane Rahmoune, Ariane Leite Rozza, Mohammed Dalli

**Affiliations:** 1 Laboratory of Integrated Cancer Research, Faculty of Medicine and Pharmacy, Hassan II University of Casablanca, Casablanca, Morocco; 2 Experimental Neurology Unit, School of Medicine and Surgery, University of Milano-Bicocca, Monza, Italy; 3 Laboratory of Health, Environment and Biotechnology, Team of Physiopathology, Molecular Genetics and Biotechnology, Faculty of Sciences Ain Chock, Hassan II University of Casablanca, Casablanca, Morocco; 4 Department of Cellular and Molecular Biology, Institute of Biosciences, São Paulo State University (Unesp), Botucatu, Brazil; 5 Higher Institute of Nursing Professions and Health Techniques, Oujda, Morocco

**Keywords:** drug resistance, flavonoids, gastroprotection, *Helicobacter pylori*, peptic ulcer disease, preclinical studies

## Abstract

Peptic ulcer disease (PUD) is a global health concern with a lifetime prevalence of 5%–10%. Current therapies may be limited by adverse effects and the increasing prevalence of drug-resistant *Helicobacter pylori* strains, highlighting the need for safer complementary approaches. Natural flavonoids have emerged as candidate bioactive compounds with gastroprotective properties in preclinical models. This systematic review, conducted according to PRISMA 2020, summarizes preclinical evidence published between 2020 and 2025 on the gastroprotective (preventive) and ulcer-healing effects of isolated and chemically characterized flavonoids. PubMed, Scopus, and ScienceDirect were searched, and eligible studies were restricted to *in vitro* and/or *in vivo* experimental investigations. Fourteen studies were included, and their risk of bias was assessed using the SYRCLE and ToxRTool tools. Gastric injury was induced mainly by ethanol, indomethacin, aspirin, or acetic acid in rats and mice, complemented by two gastric cell models. Seven of the 13 *in vivo* studies used preventive protocols, whereas only four evaluated the healing of established ulcers; the remaining studies used concurrent, post-injury, or mixed protocols. Quercetin (three studies), apigenin, and myricetin (two studies each) were the most frequently investigated, whereas luteolin, naringenin, acacetin, rhamnetin, fisetin, genistein, and malvidin were each evaluated in a single study. These compounds were associated with reduced mucosal damage and with changes in markers of oxidative stress, inflammation, apoptosis, and mucosal defense; potential anti-*H. pylori* mechanisms are discussed on the basis of previous reviews. Overall, the available evidence supports gastroprotective activity of natural flavonoids in preclinical models, whereas evidence for ulcer healing remains limited. Most studies were rated as having an unclear risk of bias, and differences in models, doses, treatment timing, and bioavailability limit direct comparison between compounds. These preclinical findings cannot be considered evidence of clinical efficacy, and pharmacokinetic, safety, and well-designed clinical studies are required before any therapeutic use in PUD can be considered.

## 1 Introduction

Peptic ulcer disease (PUD) constitutes a substantial global health burden, impacting approximately 5%–10% of the population worldwide. It is characterized by focal disruption of the mucosal lining of the stomach or duodenum (Laucirica et al., 2023; Badapanda et al., 2024; Malik et al., 2025). These lesions can result in serious complications, including obstruction, bleeding, and perforation, leading to a significant impairment in the patient’s quality of life (Gupta et al., 2025).

The pathogenesis of PUD reflects an imbalance between aggressive and protective factors in the gastrointestinal mucosa. Aggressive factors include gastric acid, pepsin, *Helicobacter pylori* infection, and exogenous insults such as the consumption of non-steroidal anti-inflammatory drugs (NSAIDs), alcohol, smoking, dietary influences, and psychological stress. In contrast, the mucosal defensive mechanisms encompass the secretion of mucus and bicarbonate, continuous epithelial regeneration, endogenous antioxidant systems, and the sustained production of cytoprotective mediators, including prostaglandin E_2_ (PGE_2_), nitric oxide (NO), sulfhydryl compounds, and growth factors (Périco et al., 2020; Akash et al., 2024). Collectively, these oxidative, inflammatory, and mucosal-defensive pathways constitute the principal mechanistic targets through which candidate gastroprotective agents, including flavonoids, are postulated to exert their pharmacological effects.

*H. pylori* infection and NSAID use are the two leading causes of PUD: *H. pylori* is implicated in approximately 90% of duodenal ulcers and 70% of gastric ulcers, whereas NSAIDs account for approximately 10%–30% of cases (Aumpan et al., 2023; Majumdar and Looi, 2024; Ko and Lee, 2025). The management of PUD primarily relies on the administration of antisecretory agents, such as proton pump inhibitors (PPIs) and histamine H_2_ receptor antagonists. These agents are often utilized in conjunction with cytoprotective compounds such as sucralfate and bismuth derivatives to enhance therapeutic efficacy. In cases associated with *H. pylori* infection, antibiotic-based eradication therapy is the cornerstone of treatment. Nevertheless, these approaches are limited by ulcer recurrence, increasing antibiotic resistance, and adverse effects associated with long-term use (Lien et al., 2022; Mustefa et al., 2023; Srivastav et al., 2023).

Therefore, complementary agents with good safety profiles are of interest for the prevention and management of PUD. In this regard, natural bioactive compounds have been widely investigated (Lien et al., 2022). Among these, flavonoids have attracted particular attention because their antioxidant, anti-inflammatory, and cytoprotective activities directly target the oxidative stress, inflammation, and impaired mucosal defense that drive ulcer formation (Chen et al., 2023; Ysrafil et al., 2023; Zhou et al., 2023; Zahra et al., 2024; Dalli et al., 2025; Pandey et al., 2025). However, this evidence remains largely experimental, and the reported effects vary considerably across models and experimental conditions.

Importantly, gastroprotection, that is, the prevention of acute mucosal injury when a compound is given before an ulcerogenic agent such as ethanol or NSAIDs, should be distinguished from ulcer healing, which requires treatment after an ulcer has been established, typically in chronic models such as acetic acid-induced ulcers (Simomura et al., 2024). These two effects involve partly different mechanisms and have different clinical implications.

A comprehensive review by (Serafim et al., 2020) summarized the gastroprotective effects of flavonoids reported between 2010 and 2020. Since then, numerous new experimental studies have been published, but this more recent evidence has not been systematically synthesized with a specific focus on individual flavonoid mechanisms or on distinguishing preventive gastroprotection from the healing of pre-existing ulcers. It remains unclear how consistently reported effects across flavonoid subclasses and experimental models support prevention of mucosal injury versus actual ulcer healing. The present review therefore examines the literature published from 2020 to 2025 on the gastroprotective (preventive) and ulcer-healing effects of isolated flavonoids in PUD, with emphasis on recent *in vitro* and *in vivo* evidence, the risk of bias of the included studies, and the underlying mechanisms, while distinguishing the predominantly preventive evidence from the considerably more limited data on the healing of established ulcers. This approach aims to clarify the current state of the evidence and identify the key gaps that should be addressed in future studies, particularly regarding the translation of experimental findings into clinical applications.

## 2 Methodology

This systematic review was conducted and reported in accordance with the PRISMA 2020 statement and aimed to evaluate the preclinical evidence on the gastroprotective effects of flavonoids in peptic ulcer disease. A literature search was conducted in PubMed, Scopus, and ScienceDirect on 27 February 2026, yielding a total of 349 records (PubMed, n = 140; Scopus, n = 122; ScienceDirect, n = 87); no additional records were identified through registers. The search strategy included combinations of the following keywords: “flavonoids,” “peptic ulcer disease,” “gastric ulcer,” “gastroprotection,” “anti-ulcer activity,” “oxidative stress,” “*H. pylori*,” “anti-inflammatory,” “*in vitro*,” and “*in vivo*,” using Boolean operators (AND, OR, NOT).

After removal of 71 duplicate records, 278 records were screened by title and abstract, of which 224 were excluded as not meeting the eligibility criteria. The remaining 54 reports were retrieved and assessed for eligibility based on full-text review. Of these, 40 were excluded for the following reasons: crude or uncharacterized plant extracts (n = 14), reviews and meta-analyses (n = 8), non-English language articles (n = 4), conference abstracts and editorials (n = 3), publication outside the 2020–2025 date range (n = 10), and non-flavonoid compounds (n = 1). Only English-language original research articles published between 2020 and 2025, restricted to *in vitro* and/or *in vivo* experimental models investigating isolated and chemically characterized flavonoids, were included; studies based exclusively on crude or uncharacterized plant extracts were excluded, in order to minimize confounding from synergistic or antagonistic interactions among multiple bioactive constituents and to allow a precise evaluation of the contribution of individual flavonoids to gastroprotection. A total of 14 studies (14 reports) were ultimately included in the review. This selection process is summarized in Figure 1. The included studies are listed in Table 1.

**FIGURE 1:**
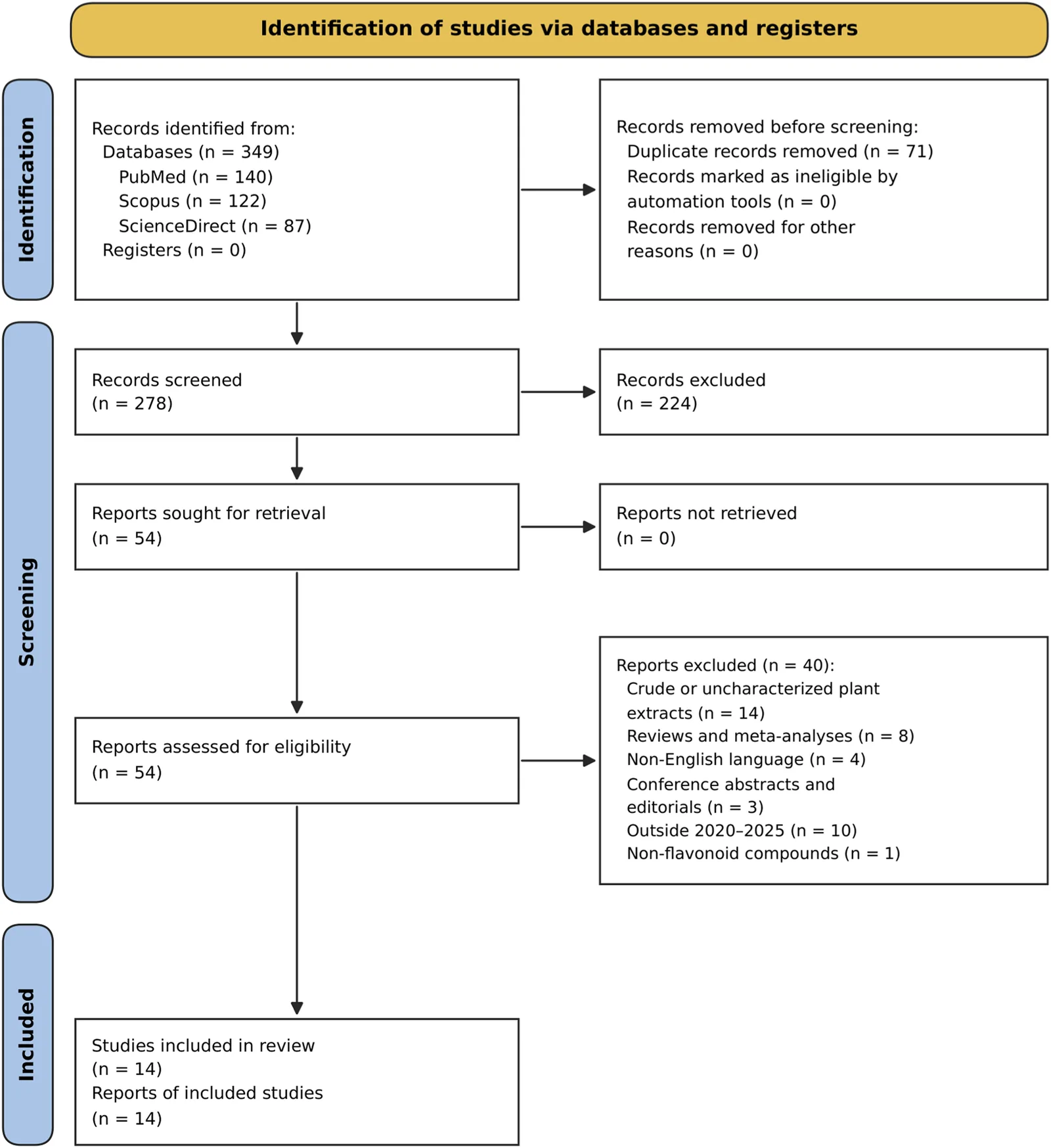
PRISMA 2020 flow diagram of the study selection process. Records were identified in PubMed, Scopus and ScienceDirect, screened by title and abstract, and assessed for eligibility based on full-text review; reasons for exclusion at the full-text stage are indicated. A total of 14 studies were included in the review.

**TABLE 1:** Protective and healing effects of flavonoids against PUD in the included preclinical studies.

Flavonoid	Subclass	Ulcer induction model	Experimental design (dose/route/duration)	Treatment protocol	Observed effects	Proposed mechanisms	References
Acacetin	Flavone	Aspirin-induced gastric ulcer (wistar albino rats)	25 mg/kg, i.p., single dose	Post-injury (2 h after aspirin)	↑TAS, ↓TOS, ↓OSI ↓TNF-α, ↓COX-2, ↓NF-κB ↑Bcl-2, ↓caspase-3	Antioxidant, anti-inflammatory, anti-apoptotic	Şimşek and Akaras, 2023
Apigenin	Flavone	Indomethacin-induced gastric ulcer (male wistar rats)	10 and 20 mg/kg, p.o., once daily for 7 days	Preventive (7 days before indomethacin)	↓Lipid peroxidation, ↑SOD, ↑CAT ↓COX-2, ↓TNF-α, ↓NF-κB ↓Bax, ↑Bcl-2, ↑TGF-β1	Antioxidant, anti-inflammatory, anti-apoptotic	Alamri, 2024
Apigenin	Flavone	Chronic acetic acid-induced gastric ulcer maintained with indomethacin (male sprague-dawley rats)	50 mg/kg, p.o., daily for 28 days	Healing (from day 5 after ulcer induction)	↑SOD, ↑CAT, ↓MDA ↑Gastric pH, ↓ulcer index ↑COX-1, ↓COX-2, ↓5-LOX	Antioxidant, cytoprotective	Abdel Fattah and Nasr El-Din, 2025
Fisetin	Flavonol	Ethanol-induced gastric ulcer (sprague-dawley rats)	25, 50 and 100 mg/kg, p.o., daily for 14 days	Preventive (14 days before ethanol)	↑SOD, ↓MDA, ↓NO, ↓MPO ↓TNF-α, ↓IL-1β, ↓IL-6 ↑PPAR-γ; ↓NF-κB and p38-MAPK signalling	Antioxidant, anti-inflammatory	Hu et al., 2020
Genistein	Isoflavone	Indomethacin-induced gastric ulcer (sprague-dawley rats)	25 mg/kg, p.o., daily for 7 days	Healing (after ulcer induction)	↓Wnt/β-catenin/TGF-β/SMAD4/PKB expression ↑SOD, ↓MDA	Anti-inflammatory, antioxidant	Hassan et al., 2023
Luteolin	Flavone	Indomethacin-induced gastric ulcer (wistar albino rats)	100 mg/kg, p.o., once daily for 28 days	Mixed (14 days before and 14 days after indomethacin)	↑SOD, ↓MDA ↑Nrf2, ↑HO-1 ↓TNF-α, ↓IL-1β	Antioxidant, anti-inflammatory	Khodir et al., 2024
Malvidin	Anthocyanidin	Ethanol-, NSAID-, ischemia-reperfusion- and acetic acid-induced ulcers (mice)	5 mg/kg, p.o	Preventive (1 h before ethanol or NSAID) and healing (acetic acid ulcer, 14 days)	↑SOD, ↑CAT, ↑GSH, ↓MDA, ↑HMOX-1 ↓TNF-α, ↓IL-6, ↑IL-10, ↓TLR4 ↑COX-1, ↓MMP-9, ↑EGF	Antioxidant, anti-inflammatory, cytoprotective, mucosal regenerative	Fagundes et al., 2021
Myricetin	Flavonol	Indomethacin-induced gastric ulcer (male wistar rats)	10 and 20 mg/kg, p.o., once daily for 14 days	Preventive (14 days before indomethacin)	↓MDA, ↑GSH, ↑SOD, ↑CAT ↓TNF-α, ↓IL-1β, ↓IL-6, ↑PGE_2_ ↓Bax, ↓caspase-3, ↓NF-κB	Antioxidant, anti-inflammatory, anti-apoptotic	Ansari et al., 2025
Myricetin	Flavonol	Ethanol-induced gastric ulcer (sprague-dawley rats)	12 mg/kg, p.o., single dose	Preventive (1 h before ethanol)	↓MDA, ↑GSSG/GSH, ↑SOD ↑COX-1, ↑PGE_2_ ↓NF-κB	Antioxidant, anti-inflammatory, cytoprotective	Park et al., 2021
Naringenin	Flavanone	Ethanol-induced gastric ulcer (BALB/c mice); ethanol-exposed KATO III gastric cells	*In vivo*: 10 and 20 mg/kg, i.g., for 3 days *In vitro*: 10 and 20 µM (2 h pretreatment)	Preventive (3 days before ethanol); *in vitro* pretreatment	↓MDA, ↓NO, ↓MPO ↓TNF-α, ↓IL-6, ↓IL-8 ↓NF-κB, ↓COX-2, ↓iNOS, ↓MAPK	Antioxidant, anti-inflammatory	Li et al., 2021
Quercetin	Flavonol	Indomethacin-induced gastric ulcer (male albino rats)	50 mg/kg, p.o., daily for 14 days	Healing (from 6 h after indomethacin)	↓Ulcer index, ↓gastric acid volume, ↑gastric pH ↓MDA, ↑SOD, ↑GSH, ↑PGE_2_, ↑NO ↓TNF-α, ↑IL-10, ↓Bax, ↑Bcl-2, ↓COX-2	Antioxidant, anti-inflammatory, anti-apoptotic, cytoprotective	Abdel-Tawab et al., 2020
Quercetin	Flavonol	Ethanol-induced gastric ulcer (wistar albino rats)	50 mg/kg, p.o., daily for 14 days	Concurrent (with ethanol)	↑CAT, ↑GSH, ↓LDH, ↓IL-6	Antioxidant, anti-inflammatory	Ahmed, 2024
Quercetin	Flavonol	TNF-α-stimulated normal human gastric epithelial cells (GES-1)	0, 0.01, 0.1, 1, 10 or 100 mM for 24 or 48 h (effective range 0.1–1 mM)	*In vitro*	↓TNF-α-induced signalling: ↓NF-κB, ↓c-Src, ↓ERK1/2, ↓c-Fos, ↓p65 ↓MMP-9 expression	Anti-inflammatory (NF-κB/MAPK inhibition), anti-invasive	Hsieh et al., 2022
Rhamnetin	Flavonol	Indomethacin-induced gastric ulcer (sprague-dawley rats)	30 and 60 mg/kg, p.o., single dose	Preventive (1 h before indomethacin)	Prevented histopathological alterations; restored gastric pH and mucin; ↓ulcer score ↑SOD, ↑CAT, ↓MDA, ↑PGE_2_ ↓TNF-α, ↓IL-6, ↑IL-10; ↑HSP70, ↓Bax	Antioxidant, anti-inflammatory, anti-apoptotic, cytoprotective	Mohammed et al., 2025

Annotation: p. o., per os; i. g., intragastric; i. p., intraperitoneal; ↑ increase; ↓ decrease. Treatment protocol: preventive, flavonoid given before the ulcerogenic agent; concurrent, given together with it; post-injury, given shortly after acute injury; healing, given after the ulcer was established.

The methodological quality of the included *in vivo* studies was assessed using the SYRCLE risk-of-bias tool for animal studies (Hooijmans et al., 2014). This tool comprises ten items covering six domains: selection bias (sequence generation, baseline characteristics, allocation concealment), performance bias (random housing, blinding of caregivers and investigators), detection bias (random outcome assessment, blinding of outcome assessors), attrition bias (incomplete outcome data), reporting bias (selective outcome reporting), and other sources of bias. Each item was rated as “low”, “high”, or “unclear” risk of bias. *In vitro* studies were appraised using the ToxRTool reliability criteria (Schneider et al., 2009). As no validated tool exists for *in vitro* efficacy studies, ToxRTool, originally designed for toxicological data, was used to describe reporting quality rather than assign a formal risk-of-bias rating. The results of the risk-of-bias assessment are summarized in Table 2.

**TABLE 2:** Risk of bias and reliability of the included studies. (A) *In vivo* studies assessed with the SYRCLE risk-of-bias tool (n = 13). (B) *In vitro* studies assessed with ToxRTool (n = 2).

Study	1. Sequence generation	2. Baseline characteristics	3. Allocation concealment	4. Random housing	5. Blinding of investigators	6. Random outcome assessment	7. Blinding of outcome assessor	8. Incomplete outcome data	9. Selective reporting	10. Other bias
Abdel Fattah and Nasr El-Din, 2025	U	L	U	U	U	U	L	U	L	U
Abdel-Tawab et al., 2020	U	L	U	U	U	U	U	U	L	U
Ahmed, 2024	U	L	U	U	U	U	U	U	L	L
Alamri, 2024	U	L	U	U	U	U	U	U	L	L
Ansari et al., 2025	U	L	U	U	U	L	L	U	L	L
Fagundes et al., 2021	U	U	U	U	U	U	U	U	L	L
Hassan et al., 2023	U	U	U	U	U	U	U	U	L	L
Hu et al., 2020	U	L	U	U	U	U	U	U	L	L
Khodir et al., 2024	U	L	U	U	U	U	U	U	L	L
Li et al., 2021	U	L	U	U	U	U	L	U	L	U
Mohammed et al., 2025	U	U	U	U	U	U	U	U	L	L
Park et al., 2021	U	L	U	U	U	U	U	U	L	L
Şimşek and Akaras, 2023	U	L	U	U	U	U	U	U	L	L

Study	Cell model	I. Test substance identification	II. Test system characterisation	III. Study design description	IV. Results documentation	V. Plausibility of design and results
Hsieh et al., 2022	GES-1 human gastric epithelial cell line	Partly (source given; purity not reported)	Partly (origin given; no authentication)	Yes	Yes (≥3 independent experiments)	Limited (0.1–1 mM, far above plasma levels; 100 mM cytotoxic)
Li et al., 2021	KATO III gastric cancer cells (ATCC)	No (source and purity not reported)	Partly (ATCC origin; no authentication)	Yes	Yes (3 independent experiments)	Yes (10–20 μM; viability checked by CCK-8)

Annotation: L, low risk of bias; H, high risk of bias; U, unclear risk of bias. Reliability categories according to Klimisch: 1, reliable without restriction; 2, reliable with restrictions; 3, not reliable.

## 3 Flavonoids: classification, sources, and general properties

### 3.1 Generalities

Flavonoids originate from the Latin word “flavus,” meaning “yellow,” and consist of a 15-carbon skeleton, presenting a C6-C3-C6 structure consisting of two benzene rings (A and B) and one heterocyclic ring C (Wei et al., 2023). The position of the B-ring on the C-ring and the number and position of hydroxyl groups on the B-ring influence the activities of flavonoids (Dias et al., 2021). Flavonoids are abundant secondary metabolites in plants, fruits, and seeds, where they contribute to color, fragrance, and flavor (Dias et al., 2021). So far, over 10,000 flavonoid compounds have been isolated and identified (Chen et al., 2023).

Among their reported pharmacological properties, flavonoids display notable antioxidant, anti-inflammatory, antiapoptotic, and cytoprotective activities (Chen et al., 2023; Ysrafil et al., 2023), which are of particular relevance to gastric mucosal protection, given the central role of oxidative stress, inflammation, and impaired mucosal defense in the pathogenesis of PUD. Antioxidant-rich plant-derived compounds, including flavonoids, have accordingly been proposed as candidate agents for the management of gastric ulcer (Altaf et al., 2023).

### 3.2 Flavonoids classification

Flavonoids are classified into several subclasses depending on their chemical structure, degree of unsaturation, and oxidation of the and oxidation of the C-ring, including flavones, flavonols, flavanols, flavanones, isoflavones, anthocyanidins, and chalcones, as shown in Figure 2. Each of these flavonoids is widely distributed in nature (Chen et al., 2023). The subclasses relevant to the flavonoids examined in this review are described below.

**FIGURE 2:**
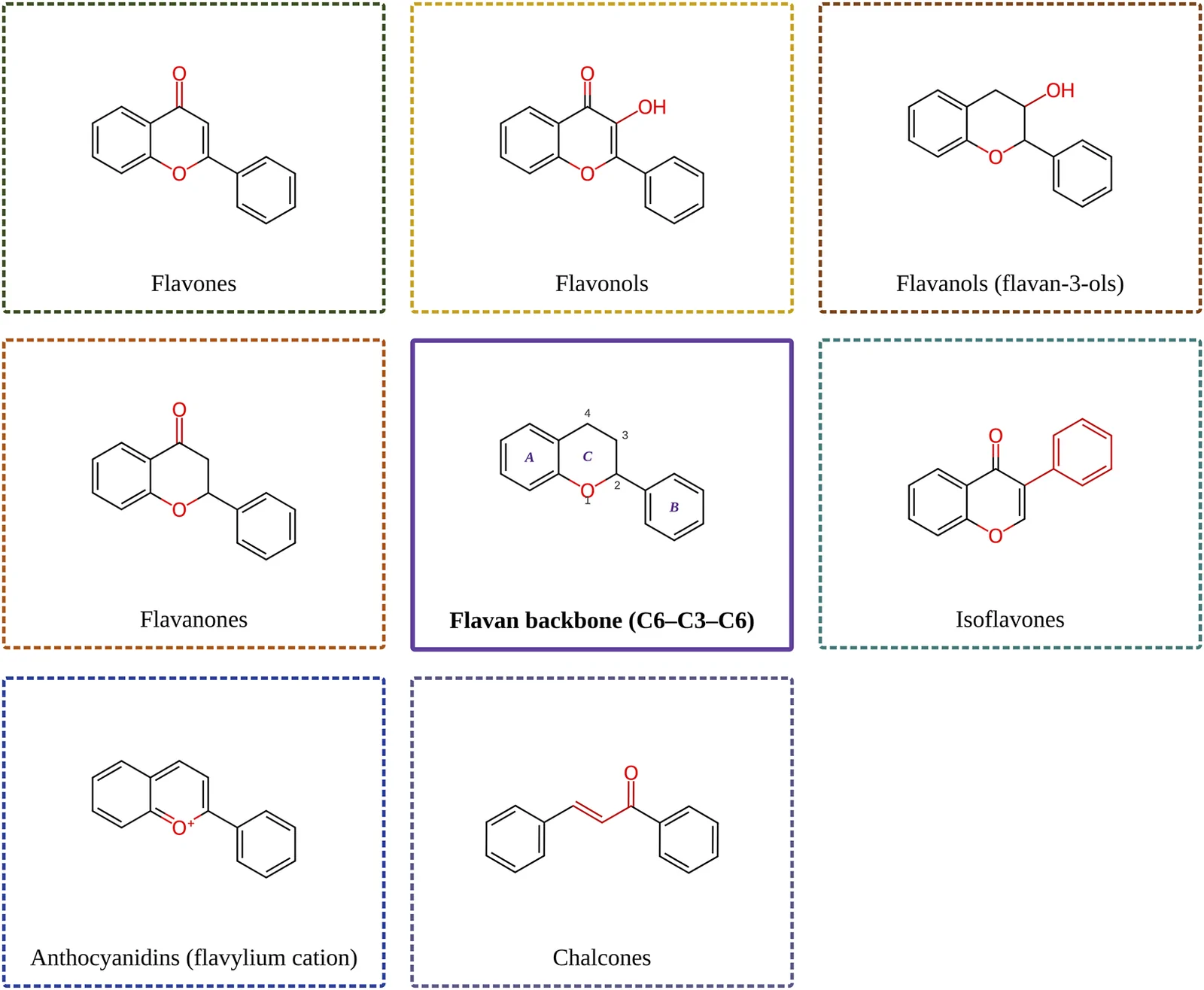
Chemical structures of the flavan backbone (C6–C3–C6; rings A, B and C) and of the main flavonoid subclasses discussed in this review. Structural features distinguishing each subclass are highlighted in red.

#### 3.2.1 Flavones

Flavones are among the most extensively studied flavonoid subclasses. Their structure consists of 4H-chromen-4-one bearing a phenyl substituent at C2, retaining the C2–C3 double bond and a C4 ketone (Chen et al., 2023). Hydroxylation typically occurs at C5 and C7 of the A-ring and at C3’ and C4’ of the B-ring, with glycosylation mainly at C7 of the A-ring; most flavones occur as 7-O-glycosides (Zhuang et al., 2023). They are widely distributed in the leaves, flowers, and fruits of many plants (e.g., celery, chamomile, mint, citrus fruits), with apigenin, luteolin, acacetin, baicalein, and chrysin among the major representatives (Zhang et al., 2020; Shen et al., 2022). Apigenin, luteolin, and acacetin, examined in this review, share notable free-radical-scavenging and antioxidant-enzyme-modulating activity, supporting their proposed cytoprotective role (Chen et al., 2023).

#### 3.2.2 Flavonols

Flavonols (3-hydroxyflavones) share the 2-phenyl-4H-chromen-4-one backbone of flavones, including the C2–C3 double bond and the C4 carbonyl group, but are distinguished by a hydroxyl group at the C3 position of the C-ring (Dias et al., 2021; Zhuang et al., 2023). Glycosylation and methylation at this position, together with O-glycosylation at C7 of the A-ring, mainly account for the structural diversity of flavonol derivatives, including kaempferol, quercetin, limocitrin, and isorhamnetin (Zhuang et al., 2023). Flavonols are among the most widespread flavonoids in the human diet, occurring in onions, kale, apples, berries and tea, with quercetin, kaempferol, myricetin and isorhamnetin as major representatives (Chen et al., 2023).

#### 3.2.3 Flavanols (flavan-3-ols)

Flavanols, or flavan-3-ols, have a saturated C-ring: they lack both the C2–C3 double bond and the C4 carbonyl group, while retaining a hydroxyl group at C3 (Dias et al., 2021; Chen et al., 2023). The two resulting chiral centres at C2 and C3 give rise to stereoisomers such as catechin and epicatechin, which may be esterified with gallic acid, as in epigallocatechin gallate. Flavan-3-ols are the monomeric units of proanthocyanidins (condensed tannins) and are abundant in tea, cocoa, grapes, apples and berries (Ramesh et al., 2021; Chen et al., 2023).

#### 3.2.4 Flavanones

Flavanones (dihydroflavones) differ from flavones by saturation of the C2–C3 bond, while retaining the C4 carbonyl group (Zhuang et al., 2023). They are typically hydroxylated at C5 and C7 of the A-ring, with O-glycosylation mainly at C7, and are predominantly distributed in citrus fruits (oranges, lemons, grapefruits, mandarins), their content varying by variety (Shen et al., 2022; Chen et al., 2023). Naringenin, the flavanone examined in this review, typically occurs as its glycoside naringin in these sources (Dias et al., 2021).

#### 3.2.5 Isoflavones

Isoflavones are distinguished by attachment of the B-ring at C3 rather than C2 of the heterocyclic C-ring, the only structural difference from flavones (Chen et al., 2023). This structure resembles 17β-estradiol, which explains their affinity for estrogen receptors and their classification as phytoestrogens (Ramesh et al., 2021; Chen et al., 2023). They occur mainly in legumes, particularly soybeans, where genistein and daidzein are the main aglycones (Dias et al., 2021; Shen et al., 2022).

#### 3.2.6 Anthocyanidins and anthocyanins

Anthocyanidins are polyhydroxylated and polymethoxylated derivatives of the flavylium (2-phenylchromenylium) cation, which, like flavan-3-ols, lacks the C4 carbonyl group; their glycosylated forms are called anthocyanins (Shen et al., 2022; Chen et al., 2023). The number and position of hydroxyl and methoxyl substituents on this flavylium core determine the individual anthocyanin species, of which more than 650 have been identified in plants to date; the six most abundant aglycones are cyanidin, delphinidin, petunidin, peonidin, pelargonidin, and malvidin (Shen et al., 2022). These water-soluble pigments are abundant in berries, grapes, and other pigmented plant tissues, and their color varies with pH (Chen et al., 2023).

#### 3.2.7 Chalcones

Chalcones (1,3-diaryl-2-propen-1-ones) re open-chain flavonoids in which the A- and B-rings are joined by a three-carbon α,β-unsaturated carbonyl bridge instead of a closed C-ring (Chen et al., 2023). They are the biosynthetic precursors of other flavonoids, and include naringenin chalcone, isoliquiritigenin, and phloretin (Ramesh et al., 2021), and exhibit a broad spectrum of pharmacological activities, including antioxidant, antibacterial, anthelmintic, antiulcer, antiviral, antiprotozoal, and anticancer effects (Shen et al., 2022). Xanthohumol and isobavachalcone are two representative derivatives that exhibit abundant biological and pharmacological activity.

Bioactivity across flavonoids is strongly governed by the number and position of hydroxyl substituents on the benzene ring, particularly the 3,5,7- and 3′,4′-dihydroxy substitution pattern (Chen et al., 2023). The most effective radical scavengers are flavonoids bearing the 3′,4′-dihydroxy pattern on the B-ring and/or a hydroxyl group at C-3; notably, the C2–C3 double bond is not required for high activity, as flavanols lacking this bond retain substantial antioxidant effects, while the presence of a 3-OH group further potentiates bioactivity (Chen et al., 2023).

## 4 Molecular mechanisms underlying flavonoid-mediated gastroprotection in *In vivo* and *In vitro* models of PUD

Experimental evidence from *in vivo* and *in vitro* studies suggests that flavonoid-mediated gastroprotection involves interconnected antioxidant, anti-inflammatory, and pro-survival signaling networks (Mustafa et al., 2026). However, only three flavonoids were evaluated in more than one study: quercetin (two *in vivo* studies and one *in vitro* study), apigenin, and myricetin (two *in vivo* studies each), whereas the remaining seven compounds were each supported by a single study. Indomethacin- and ethanol-induced acute injury were the most common models, oral doses ranged from 5 to 100 mg/kg, and only five studies tested more than one dose. Nine of the 13 *in vivo* studies included a positive control, most commonly a proton pump inhibitor. Notably, the included studies predominantly addressed preventive gastroprotection rather than the healing of established ulcers. Most used prophylactic or concurrent protocols, in which the flavonoid was given before or together with the ulcerogenic agent (Hu et al., 2020; Li et al., 2021; Park et al., 2021; Ahmed, 2024; Alamri, 2024; Khodir et al., 2024; Ansari et al., 2025; Mohammed et al., 2025). Only four studies evaluated the healing of established ulcers (Abdel-Tawab et al., 2020; Fagundes et al., 2021; Hassan et al., 2023; Abdel Fattah and Nasr El-Din, 2025). In addition, most mechanistic conclusions rely on changes in biomarker levels or protein expression measured at a single time point. Such changes are consistent with, but do not demonstrate, a causal role of the corresponding pathway; pharmacological inhibitors were used only *in vitro* (Hsieh et al., 2022). Therefore, the mechanisms described below mainly reflect the prevention of acute mucosal injury, whereas evidence for ulcer healing remains limited. The treatment protocol of each study is indicated in Table 1.

### 4.1 Modulation of oxidative stress

Oxidative stress refers to an imbalance between the excessive production of ROS and the cellular antioxidant defense system, leading to the accumulation of highly reactive molecules that damage lipids, proteins, and nucleic acids (Kıran et al., 2023). Accumulating evidence identifies oxidative stress as a central mechanism in the initiation and progression of PUD (Kıran et al., 2023; Popovic et al., 2023; Albalawi and Khateeb, 2025). Excessive ROS production induces lipid peroxidation, which compromises membrane integrity and leads to increased permeability and tissue injury, while ROS also degrade basement membrane components and cause DNA damage (Zhang et al., 2020; Gündoğdu et al., 2024). Flavonoids may limit oxidative damage both by directly scavenging ROS and by increasing the activity of endogenous antioxidant enzymes such as superoxide dismutase (SOD), catalase (CAT), and glutathione peroxidase (GPx). Reduced glutathione (GSH), in contrast, is not an enzyme but a non-enzymatic tripeptide antioxidant and the substrate used by GPx to reduce peroxides.

In the included studies, these effects were assessed indirectly by measuring antioxidant enzyme activities, GSH, and malondialdehyde (MDA), a marker of lipid peroxidation. For instance, oral quercetin (50 mg/kg for 14 consecutive days) significantly increased antioxidant enzyme activity and reduced malondialdehyde (MDA) levels, a marker of lipid peroxidation, both when administered after indomethacin-induced ulceration (Abdel-Tawab et al., 2020) and when given concurrently with ethanol (Ahmed, 2024). Similarly, other flavonoids, including rhamnetin, genistein, apigenin, and malvidin were associated with reduced MDA levels and restored SOD and CAT activities, in both preventive and healing protocols (Fagundes et al., 2021; Hassan et al., 2023; Alamri, 2024; Abdel Fattah and Nasr El-Din, 2025; Mohammed et al., 2025). In addition, myricetin pretreatment has been found to alleviate ethanol- and indomethacin-induced gastric ulcers by reinforcing endogenous antioxidant defences, reducing MDA levels and increasing PGE2 levels, thus contributing to mucosal protection against oxidative injury (Park et al., 2021; Şimşek and Akaras, 2023) also showed that acacetin, administered 2 h after aspirin, significantly increased total antioxidant status (TAS) and reduced total oxidant status (TOS) and the oxidative stress index (OSI), highlighting its ability to restore redox homeostasis. Moreover, luteolin treatment in rats not only enhanced SOD activity and decreased MDA levels but also upregulated the cytoprotective Nrf2/HO-1 pathway, a key cellular defense mechanism against oxidative stress, inflammation, and tissue damage (Khodir et al., 2024). Consistently, oral pretreatment with naringenin in BALB/c mice markedly mitigated oxidative stress, as reflected by reductions in MDA, while preserving the structural integrity of the gastric mucosa (Li et al., 2021). Likewise, fisetin and myricetin pretreatment reduced MDA levels and increased SOD and GSSG/GSH levels in ethanol-induced gastric mucosal injury (Hu et al., 2020; Park et al., 2021) (Table 1).

Beyond these conventional antioxidant responses, ferroptosis, an iron-dependent form of regulated cell death driven by lipid peroxidation and loss of GPx4 activity, has emerged as a distinct redox-related pathway. In a different disease model, the flavonol morin inhibited ferroptosis together with inflammation and oxidative stress in lipopolysaccharide-induced endometritis (Shauka et al., 2025). Because many flavonoids chelate iron and reduce lipid peroxidation, they may also interfere with ferroptosis in the gastric mucosa. However, none of the included studies assessed ferroptosis markers, and this hypothesis remains to be tested in PUD models.

### 4.2 Anti-inflammatory activity

In addition to their role in counteracting oxidative stress, flavonoids were associated with reduced gastric inflammation in experimental models, as reflected by changes in nitric oxide synthase (NOS) and myeloperoxidase (MPO) activity, inflammatory signaling pathways, and cytokine levels.

The role of NO in the gastric mucosa is context-dependent. NO produced at low levels by constitutive NOS (eNOS and nNOS) is gastroprotective: it maintains mucosal blood flow, stimulates mucus secretion, and inhibits neutrophil adhesion. In contrast, inducible NOS (iNOS), which is upregulated in neutrophils and macrophages during inflammation, produces large amounts of NO that react with superoxide to form peroxynitrite, causing oxidative damage to gastric tissues. Concurrently, neutrophil infiltration into the injured mucosa increases MPO activity, which further amplifies ROS generation (Liang et al., 2021; Lin et al., 2024).

Consistently, quercetin increased total NO levels after indomethacin injury (Abdel-Tawab et al., 2020), whereas naringenin reduced iNOS expression and NO levels after ethanol injury (Li et al., 2021), which illustrates these two opposite roles. In preventive models of ethanol-induced gastric ulcers, pretreatment with naringenin and fisetin was associated with reduced iNOS and MPO activity and lower levels of pro-inflammatory cytokines, including tumor necrosis factor-alpha (TNF-α), interleukin-8 (IL-8), and interleukin-6 (IL-6). These effects were accompanied by reduced activation of the nuclear factor kappa B (NF-κB) and mitogen-activated protein kinase (MAPK) signaling pathways (Hu et al., 2020; Li et al., 2021).

Cyclooxygenases also play opposite roles in the stomach. Constitutive COX-1 produces the prostaglandins, mainly PGE_2_, that maintain mucosal defense by stimulating mucus and bicarbonate secretion, preserving mucosal blood flow, and inhibiting acid secretion. In contrast, inducible COX-2 is upregulated during inflammation, although it also contributes to ulcer healing. NSAIDs such as indomethacin and aspirin cause ulcers mainly by inhibiting COX-1-derived prostaglandin synthesis. Three studies reported increased COX-1 expression together with higher PGE_2_ levels or enhanced mucosal repair, with myricetin, malvidin, and apigenin (Fagundes et al., 2021; Park et al., 2021; Abdel Fattah and Nasr El-Din, 2025). Downregulation of COX-2, together with reduced TNF-α and interleukin-1β (IL-1β) levels, was reported for acacetin, apigenin, quercetin, and naringenin (Abdel-Tawab et al., 2020; Şimşek and Akaras, 2023; Alamri, 2024; Abdel Fattah and Nasr El-Din, 2025; Li et al., 2025). Myricetin likewise reduced TNF-α and IL-1β levels and increased PGE_2_ (Ansari et al., 2025). *In vitro*, quercetin inhibited TNF-α-induced NF-κB and MAPK signaling and matrix metalloproteinase-9 (MMP-9) expression in gastric epithelial cells, and pharmacological inhibitors confirmed the involvement of these pathways (Hsieh et al., 2022).

Rhamnetin decreased TNF-α and IL-6 levels while increasing interleukin-10 (IL-10) levels, suggesting a potential shift toward the resolution of inflammation (Mohammed et al., 2025). Malvidin was associated with reduced toll-like receptor 4 (TLR4) expression (Fagundes et al., 2021). Genistein, given after indomethacin-induced ulceration, was associated with downregulation of the Wnt/β-catenin/TGF-β pathway and reduced mucosal fibrosis and inflammation (Hassan et al., 2023). Inflammation, oxidative stress, and ferroptosis are closely interconnected, as illustrated by the flavonol morin, which inhibited all three processes in a model of lipopolysaccharide-induced endometritis (Shauka et al., 2025), however, this evidence comes from a different disease model and its relevance to PUD remains to be established. Overall, these findings indicate that flavonoids consistently reduce inflammatory markers in experimental gastric injury, but they do not establish which pathway is responsible for the protective effect (Table 1).

### 4.3 Potential role of the gut microbiota–metabolite–mucosal barrier axis in flavonoid-mediated gastroprotection

Beyond their direct antioxidant and anti-inflammatory activities, flavonoids may contribute to gastrointestinal protection through interactions with the intestinal immune environment and mucosal integrity. Evidence from gastrointestinal inflammatory disorders indicates that modulation of inflammatory mediators and immune responses by natural compounds may help preserve mucosal homeostasis; however, findings obtained mainly in ulcerative colitis cannot be directly extrapolated to PUD and should therefore be considered as mechanistic evidence rather than clinical proof of flavonoid efficacy in PUD (Chen et al., 2024). Increasing attention has also been directed toward the intestinal microbiota, as alterations in microbial composition may influence inflammatory signaling and impair epithelial barrier function. Microbiota-directed interventions, including fecal microbiota transplantation, have demonstrated that restoring microbial communities can be accompanied by improvements in intestinal homeostasis and mucosal recovery, although therapeutic responses remain heterogeneous (Liu et al., 2021). Importantly, the effects of the microbiota may extend beyond changes in bacterial composition, since microbial metabolites such as short-chain fatty acids, particularly butyrate, can contribute to epithelial integrity and regulate local inflammatory responses. Recent findings support the close relationship between microbial composition, metabolite production, and mucosal barrier function, suggesting that this microbiota–metabolite–barrier axis may be relevant to gastrointestinal tissue repair (Liu et al., 2023; Gefen et al., 2025). Flavonoids may potentially interact with this axis by serving as substrates for microbial transformation and by influencing the abundance or activity of specific intestinal microorganisms, thereby modifying the production of bioactive microbial metabolites. Dietary glycans may further participate in this network by providing fermentable substrates for gut microorganisms and influencing their metabolic outputs. Nevertheless, these interactions remain insufficiently characterized in the context of PUD, and further experimental and clinical studies are needed to determine whether modulation of the gut microbiota and its metabolites represents a meaningful mechanism underlying the gastroprotective and mucosal-healing effects of flavonoids.

### 4.4 Anti-apoptotic activity

Apoptosis, also referred to as programmed cell death, is a rigorously regulated process that is critical for maintaining homeostasis and overall health (Li et al., 2025). Several flavonoids modulated apoptosis-related markers in experimental models of PUD. However, none of the included studies measured apoptotic cell death directly, for example, with TUNEL staining or caspase activity assays. Apoptosis was inferred from the expression of Bax, Bcl-2, caspase-3, or HSP70, measured by qRT-PCR, Western blotting, ELISA, or immunohistochemistry. In indomethacin-induced gastric injury, preventive rhamnetin increased the expression of heat shock protein 70 (HSP70) and decreased Bax expression (Mohammed et al., 2025). Similarly, quercetin, administered after indomethacin-induced ulceration, resulted in upregulation of Bcl-2 and suppression of Bax and COX-2 expression, indicating inhibition of mitochondrial-mediated apoptosis (Abdel-Tawab et al., 2020). This is the only study in which anti-apoptotic changes were observed in a healing protocol. Apigenin and myricetin, both given preventively, and acacetin, given 2 h after aspirin, were associated with increased Bcl-2 expression and reduced Bax and caspase-3 expression (Park et al., 2021; Şimşek and Akaras, 2023; Alamri, 2024; Ansari et al., 2025). Overall, these preclinical findings suggest that flavonoids protect against PUD not only by addressing oxidative stress and inflammation, but also by re-establishing the equilibrium between pro-apoptotic and anti-apoptotic factors (Table 1).

### 4.5 Anti-*Helicobacter pylori* activity

*H. pylori* is a helical-shaped Gram-negative microaerophilic bacterium that colonizes the human stomach associated with gastritis, ulcer diseases, and, in some cases, gastric cancer affecting over 40%–50% of the population from all age groups. In addition, it has been verified that this bacterial strain may also induce dyspepsia, iron deficiency anemia, and vitamin B12 deficiency (Tan et al., 2025). Infection associated with *H. pylori* is the strongest known risk factor for peptic ulcer and even gastric cancer (Zhang et al., 2020). The bacteria colonization ability relies on motility, and urease activity (Chen et al., 2025). The motility of *H. pylori* is conferred by bacterial sheathed flagella, whose filaments consist of two flagellin subunits (FlaA and FlaB), enabling the bacterium to evade recognition by TLR5 and thereby prevent activation of the host innate immune system, which is indispensable for bacterial penetration into the mucus layer (Chen et al., 2025). Moreover, *H. pylori* produces urease to maintain an alkaline environment for survival in the acidic stomach. Urease catalyzes urea into ammonia, which is toxic to intercellular junctions and causes tissue injury. Also, this type of bacteria can express adhesins, such as blood group antigen adhesin (BabA) and outer inflammatory protein adhesin (OipA), which are necessary for the attachment of these bacteria to the gastric epithelium (Zhang et al., 2020) (Figure 3, left). The ability of bacteria to adapt chemotactically relies on the pH of the gastric mucus. Adhesion ability is inseparable from adhesins, proteins anchored on the bacterial outer membrane, which are encoded by members of the large hop superfamily of outer membrane protein-encoding genes (Chen et al., 2025).

**FIGURE 3:**
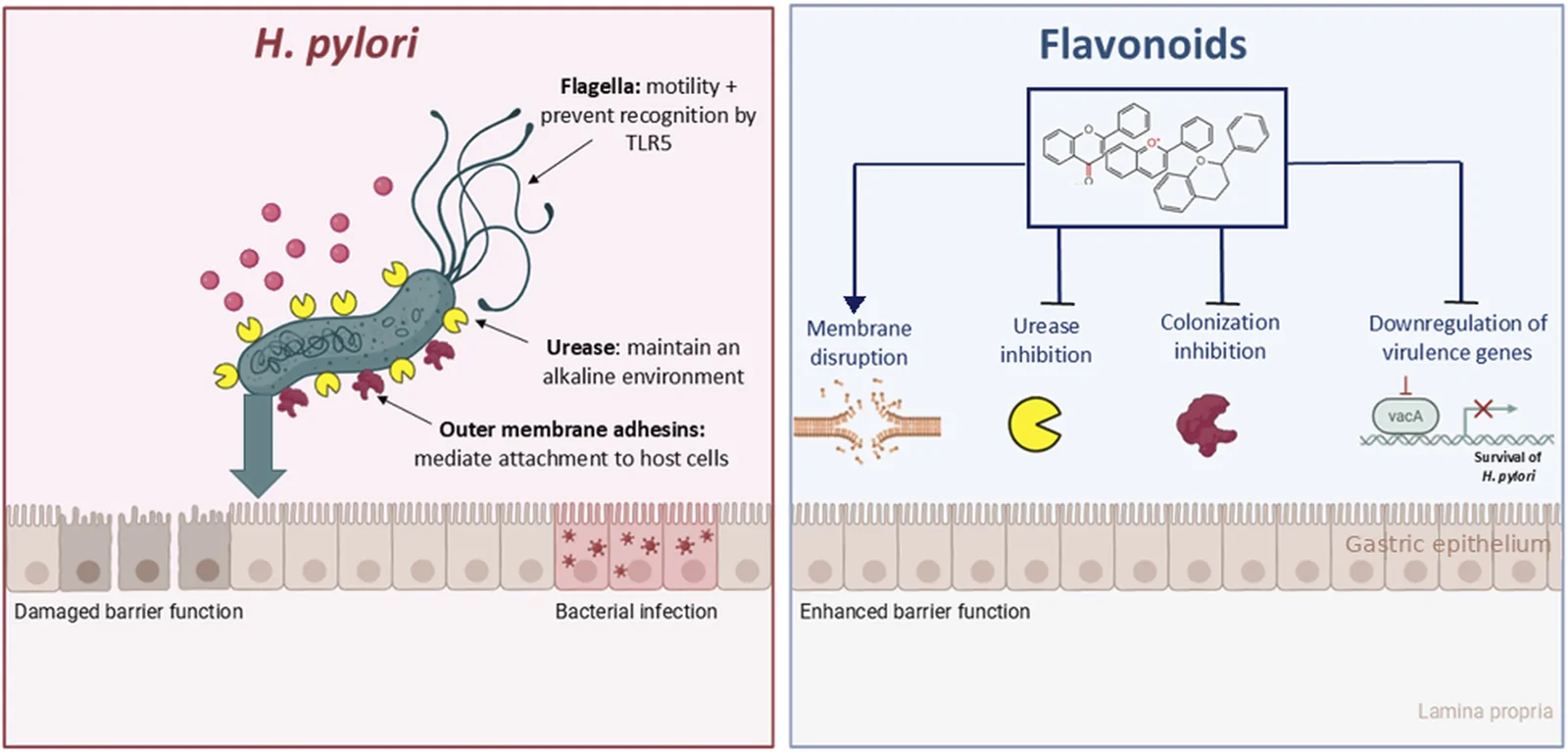
Proposed mechanisms of flavonoids against *Helicobacter pylori* infection. Left: main virulence determinants of *H. pylori* (flagella, urease and outer membrane adhesins) that enable colonization of the gastric mucosa and impair the epithelial barrier. Right: flavonoids exert anti-*H. pylori* effects by disrupting the bacterial membrane, inhibiting urease activity, inhibiting colonization, and downregulating virulence genes such as *vacA*, thereby contributing to the restoration of the epithelial barrier.

Unfortunately, current treatments for *H. pylori*-associated diseases, including antibiotic regimens, proton pump inhibitors, and bismuth-based therapies, remain limited in effectiveness, mainly because of increasing antibiotic resistance, which has stimulated interest in alternative antimicrobial strategies (Alsayeqh, 2025). Noteworthy, natural origin products contain pharmacologically active metabolites such as flavonoids, with potential antimicrobial applications (Ivyna de Araújo Rêgo et al., 2022). Further, both *in vitro* and *in vivo* studies suggested that many kinds of flavonoids possess anti-*H. pylori* activities, thus suggesting a potential contribution to the mechanisms underlying gastric protection and ulcer healing (Zhang et al., 2020) These mechanisms, which include bacterial membrane disruption, urease inhibition, inhibition of colonization, and downregulation of virulence genes, are summarised in Figure 3 (right).

As with other phytochemicals, the antimicrobial activity of flavonoids appears multifactorial, acting against different molecular targets in the pathogen instead of having one specific action site (González et al., 2021). Regarding anti-virulence effects, the hydroxyl and ketone groups in flavonoids may bind to nickel (Ni) ions or residues within the active site of the urease enzyme, thereby inhibiting its function in bacteria (Figure 3). Moreover, hydrophilic flavonoids have been suggested to interact with proteins located on the cell membrane or in the cytosol by forming complexes, thereby modulating various cellular functions, including those of adhesins, cell envelope transporters, transcriptional regulators, enzymes, and toxins (Tan et al., 2025).

According to (Ivyna de Araújo Rêgo et al., 2022), the pathogenesis of *H. pylori* depends on several virulence factors, including urease. Flavonoids can block this process once they inhibit urease activity. Flavonoids also act by inhibiting enzymes crucial for the reproduction and survival of *H. pylori*, such as DNA gyrase and ATPase. In addition, they induce ionic and metabolic imbalances at the cytoplasmic level, causing bacterial cell wall disruption, which leads to cell death or latency (Ivyna de Araújo Rêgo et al., 2022). Hence, lipophilic flavonoids, such as hesperetin, naringenin, sophoraflavanone G, and catechins with gallate groups, could penetrate the lipid bilayer membrane up to the zone under phosphate groups and laterally diffuse into the bilayer plane, causing alterations in membrane fluidity and permeability. Other flavonoids, such as quercetin, cause a decrease in the proton-motive force, impairing the production of adenosine triphosphate (ATP), while apigenin and morin induce destabilization of the membrane structure by the disordering and disorientation of membrane lipids. Also, hydrophilic flavonoids could interact at the membrane surface and/or in the cytosol with proteins involved in different essential functions. In addition, flavonoids have been reported to downregulate *H. pylori* virulence genes such as vacA, which encodes the vacuolating cytotoxin A, thereby reducing bacterial pathogenicity and survival (González et al., 2021) (Figure 3). A further benefit of flavonoids as antimicrobials against *H. pylori* infection lies in their capacity for synergism combined with anti-*H. pylori* first-line antibiotics. Notably, chrysin induced an eight-fold decrease in the MIC value of clarithromycin (CLR) (FIC = 0.125) and caused a 16-fold decrease in the MIC value of metronidazole (MTZ)(FIC = 0.0625) (González et al., 2021). These synergistic effects have so far been demonstrated only *in vitro* and have not been confirmed in animal models or in patients.

### 4.6 Limitations

Several limitations should be acknowledged. First, the internal validity of the *in vivo* studies could not be fully established: none described random sequence generation or allocation concealment, and only three reported blinded outcome assessment, so most SYRCLE domains were rated as unclear risk of bias (Table 2). The two *in vitro* studies did not report compound purity or cell line authentication, which limits the reliability of their findings (Table 2). Group sizes were small (3–10 animals, most commonly 6), and only one study reported a sample-size calculation (Khodir et al., 2024), which increases the risk of false-positive findings and overestimated effects. Only six studies assessed data normality, two did not specify the *post hoc* test (Abdel-Tawab et al., 2020; Mohammed et al., 2025), and only two used non-parametric tests for ordinal scores (Şimşek and Akaras, 2023; Alamri, 2024) (Table 3). Four studies lacked a positive control (Abdel-Tawab et al., 2020; Şimşek and Akaras, 2023; Hassan et al., 2023; Khodir et al., 2024). Third, the relevance of the doses warrants caution. Most *in vivo* doses (5–100 mg/kg, mainly oral) correspond to approximately 490–970 mg/day in a 60-kg adult for the highest doses (Reagan-Shaw et al., 2008) (Table 4). These amounts exceed dietary intake, but they fall within the range used for supplementation and may achieve relevant local concentrations in the gastric mucosa. In contrast, the 0.1–1 mM quercetin used *in vitro* (Hsieh et al., 2022) far exceeds human plasma levels, which rarely surpass 1 µM (Manach et al., 2005). Safety was formally assessed in only one study (Mohammed et al., 2025). In addition, the experimental models themselves have intrinsic limitations. Ethanol- and NSAID-induced lesions develop within hours and mainly reflect acute mucosal injury rather than chronic peptic ulcers. None of the included models involved *H. pylori* infection, the leading cause of PUD in humans, and most studies used young, healthy male rodents and short treatment periods. Chronic models such as acetic acid-induced ulcers more closely resemble human ulcers in their depth and healing process, but they were used in only two studies (de Oliveira et al., 2022). Finally, the restriction to English-language studies published between 2020 and 2025 and to isolated flavonoids may have excluded relevant work, and the heterogeneity of models and outcomes precluded a meta-analysis.

**TABLE 3:** Sample size and statistical methods reported in the included *in vivo* studies.

Study	Animals per group	Sample-size calculation	Normality test	Statistical test
Abdel Fattah and Nasr El-Din, 2025	10	No	Shapiro–Wilk	One-way ANOVA + Tukey
Abdel-Tawab et al., 2020	6	No	Not reported	One-way ANOVA + *post hoc* (not specified)
Ahmed, 2024	5	No	Not reported	One-way ANOVA + Tukey; unpaired t-test
Alamri, 2024	6	No	Kolmogorov–Smirnov	One-way ANOVA + Tukey; Kruskal–Wallis + dunn (scores)
Ansari et al., 2025	6	No	Not reported	One-way ANOVA + dunnett
Fagundes et al., 2021	3–7	No	Lilliefors	One-way ANOVA + dunnett, or Student’s t-test
Hassan et al., 2023	10	No	Kolmogorov–Smirnov	One-way ANOVA + Bonferroni
Hu et al., 2020	10	No	Not reported	One-way ANOVA + Tukey
Khodir et al., 2024	10	Yes	Shapiro–Wilk	One-way ANOVA + Tukey
Li et al., 2021	6	No	Not reported	One-way ANOVA + Tukey
Mohammed et al., 2025	6	No	Not reported	One-way ANOVA (*post hoc* not specified)
Park et al., 2021	6–7	No	Not reported	One-way ANOVA + Tukey
Şimşek and Akaras, 2023	8	No	Shapiro–Wilk	One-way ANOVA; Mann–Whitney U

**TABLE 4:** Human equivalent doses and toxicity assessment of the included *in vivo* studies.

Study	Flavonoid, dose (species)	Human equivalent dose*	Toxicity assessment
Abdel Fattah and Nasr El-Din, 2025	Apigenin, 50 mg/kg (rat)	8.1 mg/kg (∼490 mg/day)	Apigenin-only group: no adverse changes
Abdel-Tawab et al., 2020	Quercetin, 50 mg/kg (rat)	8.1 mg/kg (∼490 mg/day)	Not reported
Ahmed, 2024	Quercetin, 50 mg/kg (rat)	8.1 mg/kg (∼490 mg/day)	Not reported
Alamri, 2024	Apigenin, 10–20 mg/kg (rat)	1.6–3.2 mg/kg (∼100–195 mg/day)	Not reported
Ansari et al., 2025	Myricetin, 10–20 mg/kg (rat)	1.6–3.2 mg/kg (∼100–195 mg/day)	Not reported
Fagundes et al., 2021	Malvidin, 5 mg/kg (mouse)	0.4 mg/kg (∼25 mg/day)	Not reported
Hassan et al., 2023	Genistein, 25 mg/kg (rat)	4.1 mg/kg (∼245 mg/day)	Not reported
Hu et al., 2020	Fisetin, 25–100 mg/kg (rat)	4.1–16.2 mg/kg (∼245–970 mg/day)	General observation only (no weight loss or behavioural change)
Khodir et al., 2024	Luteolin, 100 mg/kg (rat)	16.2 mg/kg (∼970 mg/day)	Not reported
Li et al., 2021	Naringenin, 10–20 mg/kg (mouse)	0.8–1.6 mg/kg (∼50–100 mg/day)	Not reported *in vivo* (cell viability checked *in vitro*)
Mohammed et al., 2025	Rhamnetin, 30–60 mg/kg (rat)	4.9–9.7 mg/kg (∼290–580 mg/day)	Acute toxicity test: no toxic signs at 400 mg/kg
Park et al., 2021	Myricetin, 12 mg/kg (rat)	1.9 mg/kg (∼115 mg/day)	Not reported
Şimşek and Akaras, 2023	Acacetin, 25 mg/kg i.p. (rat)	4.1 mg/kg†	Not reported

Annotation: *: Calculated by body surface area normalization (Reagan-Shaw et al., 2008) for a 60-kg adult (rat dose × 0.162; mouse dose × 0.081). †Intraperitoneal administration, not directly comparable with oral intake.

Overall, these findings derive exclusively from animal and cell models and cannot be directly extrapolated to humans. No flavonoid reviewed here is approved for peptic ulcer disease, and well-designed clinical trials remain essential.

## 5 Conclusion

In this review, we presented an overview of the current preclinical research on the gastroprotective potential of natural flavonoids in peptic ulcer disease. We summarized their classification, major natural sources, and the main biological activities that may contribute to their protective effects on the gastric mucosa. The available evidence, derived from 14 studies on ten isolated flavonoids, consistently shows that flavonoids reduce acute gastric mucosal injury when administered before or together with an ulcerogenic agent, mainly in ethanol- and NSAID-induced models. In contrast, evidence for the healing of established ulcers is limited to four studies, and only quercetin, apigenin, and myricetin have been evaluated in more than one study. The protective effects of flavonoids were associated with changes in multiple pathways involved in oxidative stress, inflammation, apoptosis, and mucosal defense, rather than with a single mechanism; however, these associations were rarely confirmed by direct mechanistic experiments. Anti-*H. pylori* activity, although promising, is supported mainly by *in vitro* data from previous studies. Furthermore, most included studies had an unclear risk of bias and small sample sizes, and the effective doses often exceeded dietary intake. Therefore, these findings support flavonoids as candidates for further investigation rather than as therapeutic agents for PUD. Future studies should prioritize chronic ulcer-healing models, dose–response and pharmacokinetic evaluations, formal toxicity assessment, and rigorous study designs with randomization and blinding, before well-designed clinical trials can be considered.
